# Fig Leaf Bioactivity and Safety: Temperature Optimization and FTIR Authentication

**DOI:** 10.1002/fsn3.71508

**Published:** 2026-02-04

**Authors:** Ekarat Vasupen, Kanokkarn Rabpairee, Watcharaporn Toommuangpak, Onpilin Sompeerapun, Utumporn Chaiwong, Phattharaporn Yuthachit, Natta Kachenpukdee, Siriwan Nawong, Numphon Thaiwong

**Affiliations:** ^1^ Faculty of Agricultural Innovation and Technology Rajamangala University of Technology Nakhon Ratchasima Thailand; ^2^ Synchrotron Light Research Institute (Public Organization) Nakhon Ratchasima Thailand; ^3^ Faculty of Agriculture National Corn and Sorghum Research Center, Kasetsart University Nakhon Ratchasima Thailand; ^4^ Food Technology Program, Faculty of Science and Technology Nakhon Ratchasima Rajabhat University Nakhon Ratchasima Thailand; ^5^ Aquaculture and Fishery Product Department, Faculty of Science and Fisheries Technology Rajamangala University of Technology Srivijaya Trang Thailand

**Keywords:** drying temperature optimization, fig leaves, FTIR fingerprinting, safety validation, selective cytotoxicity

## Abstract

*Ficus carica* L. leaves represent an underutilized agricultural byproduct despite growing consumer interest in functional foods. Four fig leaf cultivars representing diverse geographic origins (BTM, Black Violet, Longue d'Aout, and Sultane) were compared to investigate drying temperature (50°C–80°C) effects on bioactivity through water extraction. The extract demonstrating superior antioxidant activity was subsequently evaluated for safety using cell‐based cytotoxicity testing. Bioactive profiling assessed total phenolic content (TPC) and total flavonoid content (TFC). Fourier‐transform infrared (FTIR) spectroscopy with principal component analysis (PCA) accomplished cultivar discrimination. Cell‐based cytotoxicity testing via 3‐(4,5‐dimethylthiazol‐2‐yl)‐2,5‐diphenyltetrazolium bromide (MTT) assay evaluated safety on Caco‐2, HepG2, and THLE‐2 cells. Results identified 60°C as the optimal drying temperature across all cultivars (*p* < 0.05). Longue d'Aout demonstrated superior bioactivity: TPC = 53.8 mg GAE/g extract, DPPH (2,2‐diphenyl‐1‐picrylhydrazyl) IC_50_ = 0.96 mg/mL. Higher temperatures (70°C–80°C) significantly reduced bioactivity. Conversely, ABTS (2,2′‐azino‐bis(3‐ethylbenzothiazoline‐6‐sulfonic acid)) and FRAP (ferric reducing antioxidant power) revealed cultivar‐specific temperature responses. FTIR‐PCA successfully discriminated cultivars with 96.8% accuracy (PC‐1: 85%, PC‐2: 7%). All extracts demonstrated excellent safety (IC_50_ = 7–15.3 mg/mL, safety factor 70–1530×). Selective cytotoxicity to cancer cells emerged: HepG2 (IC_50_ = 7 mg/mL) versus hepatocytes THLE‐2 (IC_50_ = 15.3 mg/mL), showing 2.18‐fold selectivity. FTIR achieved 96.8% discrimination accuracy for quality control. Water‐based extraction assessment confirmed excellent safety profiles in normal hepatocytes and selective cancer cell toxicity. Superior bioactivity and excellent safety profiles validate fig leaf extracts as safe functional food ingredients, warranting investigation into their potential anti‐cancer mechanisms.

## Graphical abstract

Optimization of Fig Leaf Processing for Functional Food Applications 60°C represents the optimal drying temperature for preserving bioactive compounds. Among four fig cultivars (BTM, Sultane, Black Violet, and Tongue d’Aout), Tongue d’Aout demonstrates superior bioactive retention. FTIR spectroscopy achieves 92% cultivar discrimination accuracy. Water extraction provides a sustainable and clean‐label ready approach for functional food ingredient development.

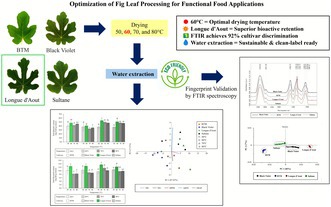

## 1 Introduction

Changing demographic trends and rising health awareness are driving the expansion of the global functional food market (Ahmed et al. 2025). Consumers increasingly seek health‐promoting ingredients in daily food products with scientifically validated health benefits. Food industry responses focus on developing novel ingredients from underutilized plant resources to meet growing demand for functional foods. Such ingredients offer several advantages, including natural origin, consumer acceptance, and potential synergistic bioactive effects.

*Ficus carica* L. (fig), a plant native to southwest Asia and the Middle East, is now cultivated worldwide across diverse climatic regions (Bao et al. 2023; Gündeşli et al. 2024; Zahid et al. 2025). Global dried fig production reached 1.26 million tons in 2022, with Turkey dominating production, representing nearly 25% of worldwide output (Ramadan 2023). Although fig fruits remain the primary commercial product, fig leaves constitute valuable agricultural byproducts generated during fruit harvesting and processing. Developing fig leaf ingredients aligns with sustainable food systems while generating value‐enhanced functional materials from otherwise underutilized resources (Diaz‐Ambrona and Maletta 2014). Fig leaves contain three major classes of phenolic compounds: anthocyanins, flavonols, and rutin, each with distinct chemical structures and biological functions. Anthocyanins represent water‐soluble flavonoid pigments possessing potent antioxidant and anti‐inflammatory properties (Li et al. 2021). Flavonols, including quercetin and luteolin, exhibit strong free radical‐scavenging capacity via their aromatic ring systems (Tikent et al. 2024). Rutin demonstrates enhanced bioavailability, enabling effective absorption into human tissues (Winanta et al. 2025). Combined antioxidant properties support multiple human health outcomes, such as cardiovascular health, metabolic function, and anti‐inflammatory responses (Li et al. 2021; Tikent et al. 2024). Clinical evidence supports fig leaf therapeutic applications for managing blood glucose levels, allergic responses, and dermatological conditions (Abe et al. 2022). Traditional medicine systems have utilized fig leaves for centuries, providing an ethnopharmacological foundation for modern scientific validation of bioactivity claims.

Drying represents a standard processing method for preparing agricultural plant materials due to practical simplicity and effectiveness. Processing methods significantly affect bioactive compound retention in raw plant materials, with processing temperature representing a critical variable (Cilla et al. 2018). Drying temperature critically influences the retention and bioavailability of bioactive compounds, directly affecting the quality of functional food ingredients (ElGamal et al. 2023). Elevated temperatures generally cause degradation of phenolic compounds and loss of antioxidant activity through thermal decomposition and oxidation reactions (Antony and Farid 2022). Plant species and cultivars respond differently to temperature variations, reflecting their distinct chemical compositions and thermal stability. Olive leaves treated at 105°C exhibited substantial phenolic losses compared to 60°C treatment, losing approximately 40%–60% of phenolic compounds (Kamran et al. 2015). *Myrtus communis* leaves demonstrated superior extraction efficiency at 70°C compared to higher temperatures, indicating optimal processing windows vary by species (Snoussi et al. 2022). Fig exhibits exceptional genetic diversity encompassing over 800 recognized species and cultivars (Bao et al. 2023). Each cultivar potentially requires distinct processing parameters reflecting its unique chemical composition and thermal stability. Water extraction represents a promising approach for recovering phenolic compounds and bioactive compounds suitable for beverage applications and functional food formulations (Plaskova and Mlcek 2023). The effectiveness of water‐based extraction stems from its ability to avoid concerns about organic solvent toxicity while maintaining compliance with food processing regulations and consumer preferences. Multiple advantages justify selecting water extraction as the primary extraction method. Knowledge gaps remain regarding optimal cultivar selection and the identification of processing temperatures necessary to maximize functional ingredient development. Identifying appropriate cultivar‐processing combinations is essential to establishing efficient protocols for producing fig leaf functional food ingredients. Addressing knowledge gaps about cultivar‐temperature optimization will enable comprehensive ingredient development strategies.

FTIR spectroscopy combined with multivariate analysis provides rapid, non‐destructive characterization of plant materials, enabling cultivar discrimination and quality assurance through unique chemical fingerprints (Zhong et al. 2024). FTIR spectroscopy offers advantages over chromatographic approaches, including faster analysis and minimal sample preparation (Zhong et al. 2024). While such analytical methods characterize chemical composition, traditional antioxidant assays (DPPH, ABTS, FRAP) assess radical scavenging capacity. Yet, comprehensive evaluation of functional food ingredients requires direct cytotoxicity assessment on human cells. Cell‐based assays using multiple tissue types (intestinal, hepatic, normal hepatic) enable tissue‐specific safety evaluation and assessment of selective responses to cancer cells.

Fig leaves represent an underutilized resource with insufficient knowledge regarding temperature‐dependent bioactivity and cultivar variation. This study investigated four cultivars (BTM, Black Violet, Longue d'Aout, Sultane) with four complementary objectives: (1) investigate drying temperature effects (50°C–80°C) on bioactive retention, (2) employ FTIR‐PCA for cultivar characterization, (3) evaluate selective cytotoxicity using multiple cell lines, and (4) identify optimal cultivar‐processing combinations. The integrated approach addresses critical knowledge gaps and provides scientific evidence for fig leaf development as functional food ingredients.

## 2 Materials and Methods

### 2.1 Experimental Overview

**FIGURE 1:**
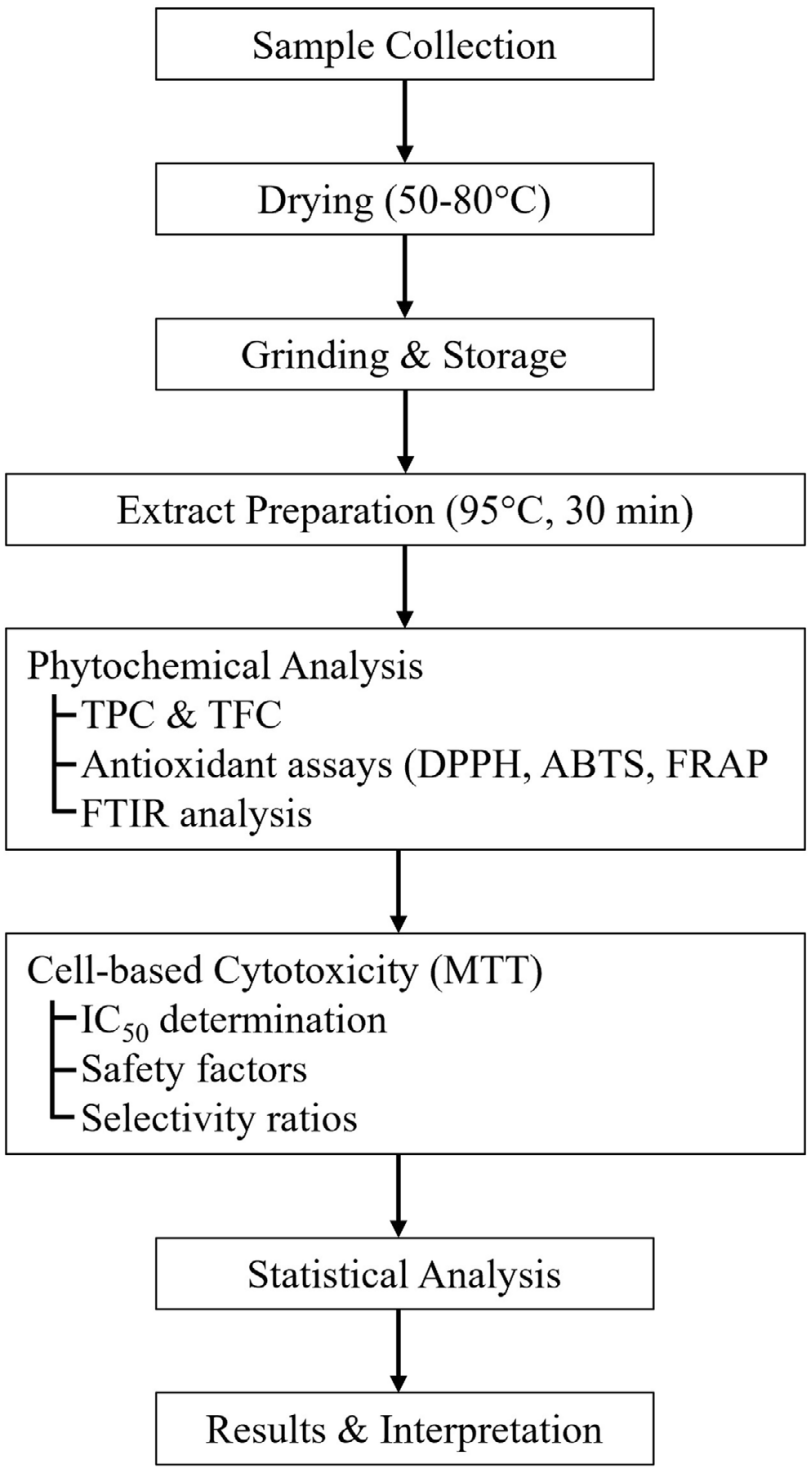
Experimental workflow for phytochemical profiling and antioxidant assessment of fig leaf extracts. The diagram illustrates the sequential progression from sample preparation through final results interpretation.

An overview of the complete experimental workflow is presented in Figure 1. The methodology comprises six major stages: (1) sample collection, air‐drying, grinding, and storage, (2) aqueous extraction at optimized conditions, (3) phytochemical analysis including TPC, TFC, and antioxidant activity assessment, (4) spectroscopic analysis by FTIR, (5) cell‐based cytotoxicity evaluation, and (6) statistical analysis and results interpretation. Detailed descriptions of each experimental stage are provided in the following sections.

### 2.2 Plant Materials

Four *F. carica* L. cultivars were selected: BTM (Brown Turkey Modified), a high‐yielding cultivar (Stover et al. 2007), Black Violet, containing high anthocyanins (Solomon et al. 2006), Longue d'Aout, a traditional French cultivar (Flaishman et al. 2008), and Sultane, valued for superior quality (Simonet et al. 1945). These cultivars exhibited diverse leaf morphology, from deeply lobed (Black Violet, Longue d'Aout) to moderately dissected forms (BTM, Sultane).

### 2.3 Sample Collection and Processing

Leaves at the maturity stage were obtained from fig trees planted in Pak Chong District, Nakhon Ratchasima Province, Thailand. The second leaf from the apex was harvested from each tree to ensure physiological maturity and consistent phytochemical composition. Each tree served as an independent biological replicate. Fresh leaves were washed, drained, and cut into 1 × 1 cm pieces. Tray drying was performed at four temperatures (50°C, 60°C, 70°C, or 80°C) for 12 h to achieve a moisture content below 8%. Dried materials were ground using a laboratory mill (Retsch ZM 200, Retsch GmbH, Haan, Germany) for 30 s at 20,000 rpm, passed through a 60‐mesh sieve (250 mm), and stored in aluminum foil pouches within desiccators containing silica gel at room temperature (25°C ± 2°C) until analysis.

### 2.4 Chemicals and Reagents

All chemicals used were of analytical grade. Folin–Ciocalteu reagent, 2,2‐diphenyl‐1‐picrylhydrazyl (DPPH), 2,2′‐azino‐bis (3‐ethylbenzothiazoline‐6‐sulfonic acid) (ABTS), and 2,4,6‐tripyridyl‐s‐triazine (TPTZ) were purchased from Sigma‐Aldrich (St. Louis, MO, USA). Gallic acid, quercetin, and Trolox standards were also obtained from Sigma‐Aldrich. Sodium carbonate (Na_2_CO_3_), aluminum chloride (AlCl_3_), potassium acetate (CH_3_COOK), ferric chloride (FeCl_3_), and other routine chemicals were purchased from Loba Chemie (Mumbai, India).

### 2.5 Preparation of Fig Leaf Extract

Extraction conditions were optimized based on preliminary studies (Bao et al. 2023). Fig leaf extracts were prepared by mixing 1.0 g of dried powder with 25 mL of deionized water (1:25 w/v) and allowing the mixture to extract for 30 min. Extraction was performed at 95°C using a shaking water bath (WNB 14, Memmert GmbH, Schwabach, Germany). The extraction time was balanced to achieve compound recovery while maintaining thermal stability. Following cooling to room temperature for 15 min, the solution was passed through qualitative filter paper (Grade 2, Whatman International Ltd., Maidstone, UK). The filtrate underwent rotary evaporation at 40°C (Büchi Rotavapor R‐300, BÜCHI Labortechnik AG, Flawil, Switzerland). Concentration continued under reduced pressure until complete dryness was achieved. The concentrated extract was stored at −20°C until analysis. The extract was dissolved in deionized water to achieve the desired assay concentrations.

### 2.6 Determination of Total Phenolic Content (TPC)

TPC was assessed using the modified Folin–Ciocalteu procedure (Singleton et al. 1999) with gallic acid standards (0–100 μg/mL). A sample extract (0.5 mL) was combined with Folin–Ciocalteu reagent (2.5 mL, 1:1 v/v) and incubated for 3 min. The mixture was then mixed with 7.5% Na_2_CO_3_ (2.0 mL) and incubated in the dark for 60 min. Subsequently, the reaction solution was analyzed for absorbance at 765 nm (UH5300, Hitachi High‐Tech Corporation, Tokyo, Japan), and data were presented as mg gallic acid equivalents (GAE)/g extract.

### 2.7 Determination of Total Flavonoid Content (TFC)

Total flavonoid content was assessed through the aluminum chloride colorimetric assay (Chang et al. 2002). A sample extract (1.5 mL) was mixed with deionized water (2.8 mL), 10% w/v AlCl_3_solution (100 μL), and 1M CH_3_COOK solution (100 μL), and then incubated for 30 min before measuring the absorbance at 420 nm. Results were calculated using quercetin standards (0–100 mg/L) and expressed as mg QE/g extract.

### 2.8 DPPH Radical Scavenging Assay

The DPPH radical scavenging capacity was evaluated following the procedure of Brand‐Williams et al. (1995). Extract solutions at various concentrations (0.1 mL) were mixed with DPPH solution (2.9 mL, 0.1 mM in ethanol), kept in darkness for 30 min, and measured at 517 nm. Inhibition percentages were computed using Equation (1):

(1) $$\%\text{Inhibition}=\left[\left(A_{0}-A_{1}\right)/A_{0}\right]\times 100$$

where *A*_0_ represents the absorbance of the negative control (DPPH + ethanol) and *A*_1_ represents the absorbance of the test sample (DPPH + extract).

The IC_50_ value (concentration required to scavenge 50% of DPPH radicals) was determined by plotting the inhibition percentage against extract concentration and calculating the concentration at 50% inhibition using linear regression analysis. Gallic acid was used as a positive control. Results were expressed as IC_50_ values (μg/mL).

### 2.9 ABTS Radical Scavenging Assay (ABTS)

ABTS radical scavenging activity was assessed following the procedure of Re et al. (1999). ABTS radical cation was generated by mixing ABTS with K_2_S_2_O_8_ (1:1 v/v) and incubating for 12–16 h, then diluted to achieve an absorbance of 0.700 ± 0.05 at 734 nm. A sample extract (20 μL) was mixed with an ABTS solution (1.98 mL), incubated for 5 min, and measured at 734 nm. Data were presented as mg TE/100 g extract using Trolox standards.

### 2.10 Ferric Reducing Antioxidant Power Assay (FRAP)

FRAP activity was quantified according to Benzie and Strain (1996). The assay was performed using fresh working reagent containing sodium acetate buffer (pH 3.6), FeCl_3_, and TPTZ at a 10:1:1 ratio. A sample extract (150 μL) was mixed with FRAP reagent (1.5 mL), incubated for 20 min, and measured at 593 nm. Values were reported as mg TE/100 g extract based on Trolox standards.

### 2.11 Fourier‐Transform Infrared (FTIR) Spectroscopy Analysis

Sample preparation for FTIR analysis used the drop‐coating approach. Extract solutions (5–10 μL) were deposited on barium chloride (BaCl_2_) optical windows and vacuum‐dried at room temperature, ensuring uniform distribution and enhanced spectral signal intensity. Infrared spectroscopic measurements were conducted using a Hyperion 2000 Infrared Microscope (Bruker Optics, Ettlingen, Germany) equipped with a mercury cadmium telluride (MCT) detector in transmission mode. Spectra were acquired in the wavenumber region of 4000–600 cm^−1^ with spectral resolution of 4 cm^−1^, 64 co‐averaged scans per spectrum, 15× magnification objective lens, and 50 × 50 μm^2^ detector aperture. For each cultivar, at least 50 spectral measurements were acquired from different sample positions. Absorbance intensity values were maintained below 1.0 to ensure optimal signal‐to‐noise ratios. Data collection was performed using OPUS 7.8 software (Bruker Optics, Ettlingen, Germany).

Spectral preprocessing included baseline correction using the asymmetric least squares algorithm and vector normalization to account for sample thickness variations. Peak intensity ratios were extracted and normalized to an internal standard for quantitative comparison across cultivars. Multivariate spectral analysis using the Unscrambler X 10.5 software (CAMO Analytics, Oslo, Norway) performed Principal Component Analysis (PCA) for cultivar discrimination based on unique phytochemical profiles. FTIR‐PCA discriminatory power was validated through Leave‐One‐Out Cross‐Validation (LOOCV). For each spectrum from the dataset (*n* = 200, 50 per cultivar), it was sequentially removed, and a new PCA model was built from the remaining 199 spectra. Classification accuracy was calculated as the percentage of correctly classified spectra.

### 2.12 Cell‐Based Cytotoxicity Assessment

The cell‐based cytotoxicity testing was performed using three distinct human cell lines representing different tissue types: intestinal epithelial cells (Caco‐2), hepatic cancer cells (HepG2), and normal hepatocytes (THLE‐2) to establish the safety profile of fig leaf extracts for human consumption. This multi‐cell‐line approach allowed simultaneous assessment of (1) potential toxicity to normal cells, (2) selective responses to cancer cells, and (3) tissue‐specific cytotoxic patterns.

Three human cell lines were used in this study: Caco‐2 (human colorectal adenocarcinoma), HepG2 (human hepatocellular carcinoma), and THLE‐2 (human normal liver epithelial cells). Cell lines were propagated in Dulbecco's Modified Eagle Medium (DMEM) enriched with 10% FBS, penicillin (100 U/mL), and streptomycin (100 mg/mL), employing the culturing methods outlined by Thusyanthan et al. (2022). Cells were cultured at 37°C in a humidity‐controlled environment containing 5% CO_2_. Fresh medium was supplied every 3 days, and cell subculturing was initiated when cultures reached 80% confluence via treatment with 0.25% trypsin–EDTA.

Cell viability and cytotoxic potential of fig leaf extracts were evaluated using the 3‐(4,5‐dimethylthiazol‐2‐yl)‐2,5‐diphenyltetrazolium bromide (MTT) assay following recent comprehensive methodology guidelines (Ghasemi et al. 2023). Briefly, cells (5000 cells/well) were seeded into sterile 96‐well plates (100 mL/well) and incubated for 24 h to allow cell attachment. Fig leaf extracts (Longue d'Aout cultivar, prepared at 60°C optimal drying temperature) were dissolved in serum‐free DMEM and tested at concentrations ranging from 0.0001 to 10 mg/mL. After 48 h exposure to extract concentrations at 37°C in 5% CO_2_, cells were treated with 10 mL of MTT solution (5 mg/mL) and incubated for 4 h at 37°C (Thusyanthan et al. 2022). Formazan crystals (purple) were solubilized in 100 mL of dimethyl sulfoxide (DMSO), and the absorbance of the resulting solutions was measured at 570 nm using a Tecan Sunrise microplate reader (Switzerland). Cell viability was calculated as Equation (2):

(2) $$\begin{array}{cc} \text{Cell Viability}\;(\%)=\; & (\text{Absorbance of treated cells}/\text{Absorbance of }\\ & \text{control cells})\times 100 \end{array}$$

where control cells receive equivalent volumes of vehicle (DMSO) without test compounds.

IC_50_ values were derived from non‐linear regression analysis using GraphPad Prism (Version 8.0) based on established guidelines (Ghasemi et al. 2023). Nontoxic concentration thresholds were defined as ≥ 90% cell viability, and safety factors were calculated as the ratio of IC_50_ to practical consumption concentration (0.01–0.1 mg/mL). Selectivity ratios were calculated as the ratio of IC_50_ values for normal hepatocytes (THLE‐2) to those for cancer cells (HepG2). Triplicate assays (*n* = 3) were conducted, and findings were expressed as mean ± SD. A one‐way ANOVA with Tukey's post hoc test was used (*p* < 0.05).

### 2.13 Statistical Analysis

Data were analyzed using a factorial completely randomized design with three technical replications and expressed as the mean ± SD (*n* = 3). ANOVA followed by Duncan's test (*p* < 0.05) evaluated temperature and cultivar effects using SPSS 29.0 (IBM Corp., Armonk, NY, USA). Pearson's correlation analysis was performed to assess relationships among phytochemical parameters and antioxidant activities using SPSS 29.0. Principal component analysis (PCA) of biochemical parameters was conducted using PAST 4.13 (Paleontological Statistics Software Package, Natural History Museum, University of Oslo, Norway) to analyze variable relationships and cultivar discrimination.

## 3 Results and Discussion

### 3.1 Morphological Characteristics of Fig Cultivars

**FIGURE 2:**
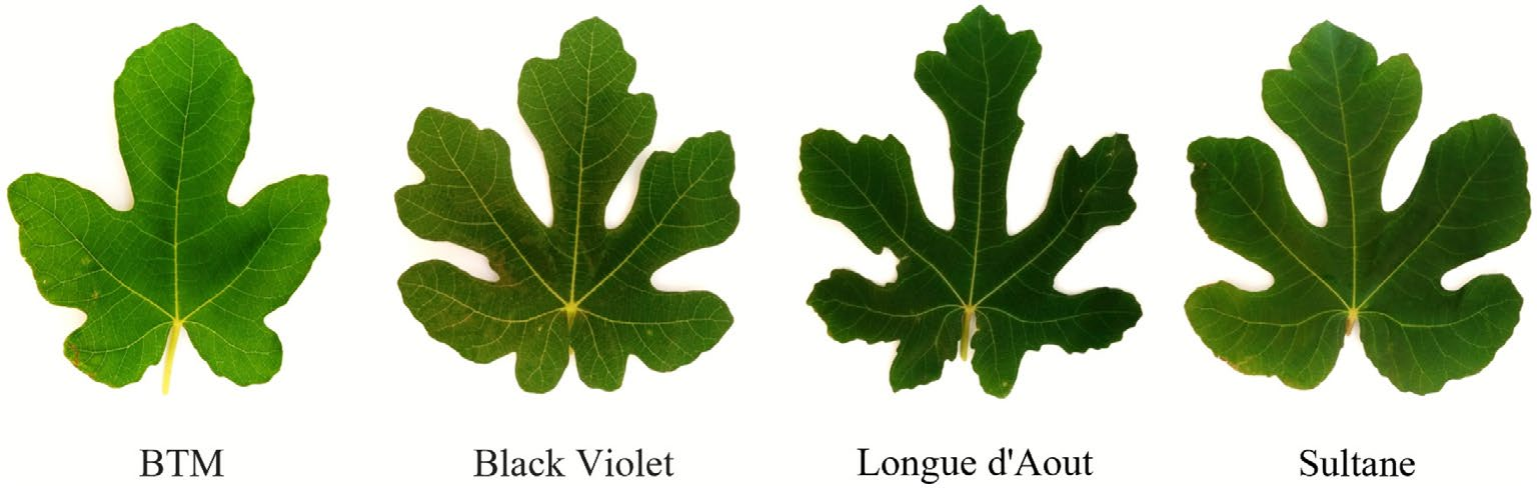
Morphological characteristics of fresh leaves from four fig ( *F. carica* L.) cultivars: BTM, Black Violet, Longue d'Aout, and Sultane, showing diverse leaf shapes and lobing patterns. Scale bar = 5 cm.

The four fig cultivars exhibited distinct leaf morphology (Figure 2), ranging from deeply lobed leaves in Black Violet and Longue d'Aout to moderately dissected forms in BTM and Sultane. These morphological differences may correlate with their phytochemical profiles.

### 3.2 Total Phenolic Content and Total Flavonoid Content

**FIGURE 3:**
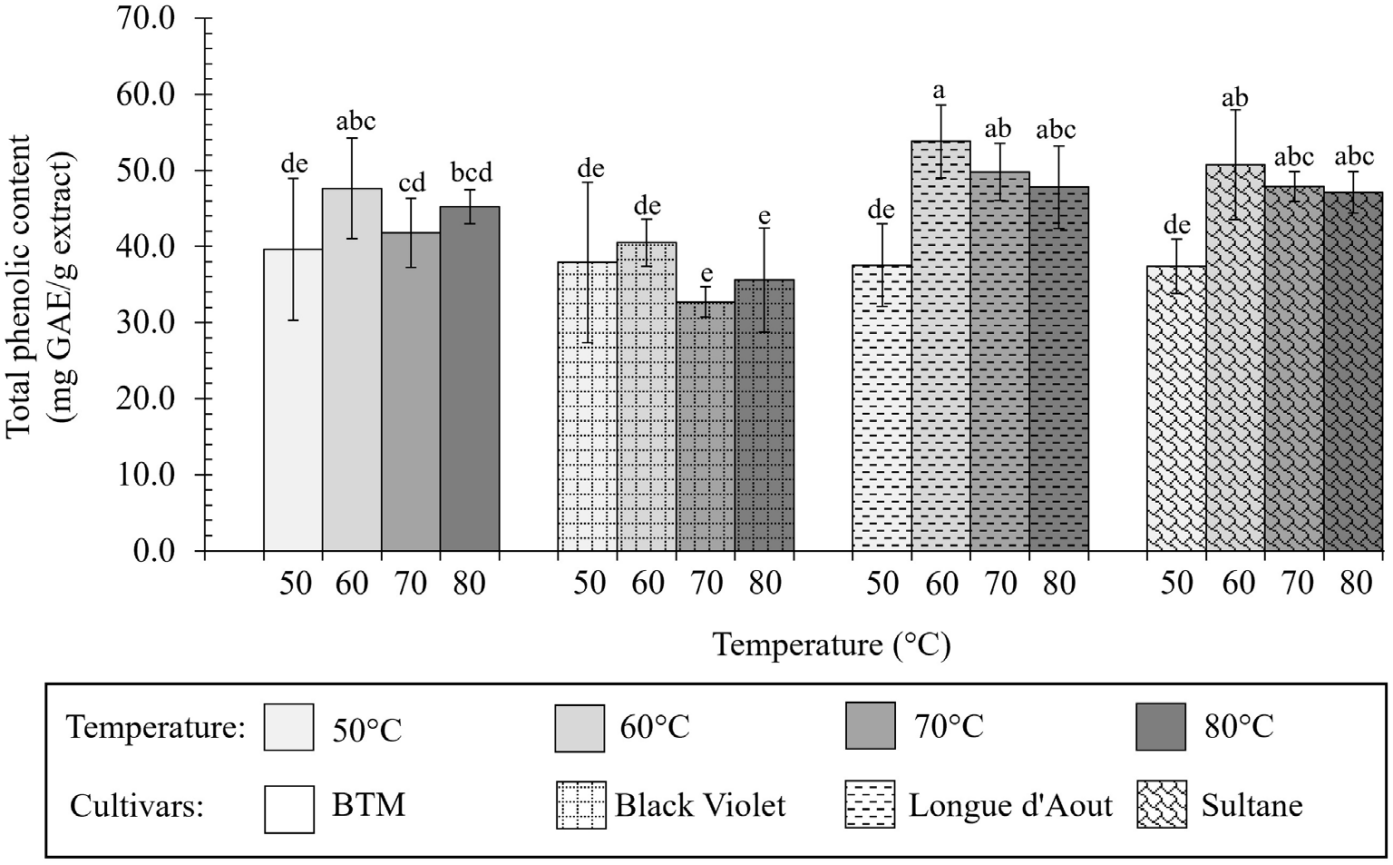
Impact of drying temperature on total phenolic content (mg GAE/g extract) of fig leaf extracts from different cultivars using water extraction. Different letters indicate significant differences (*p* < 0.05). Data represent mean ± SD (*n* = 3).

**FIGURE 4:**
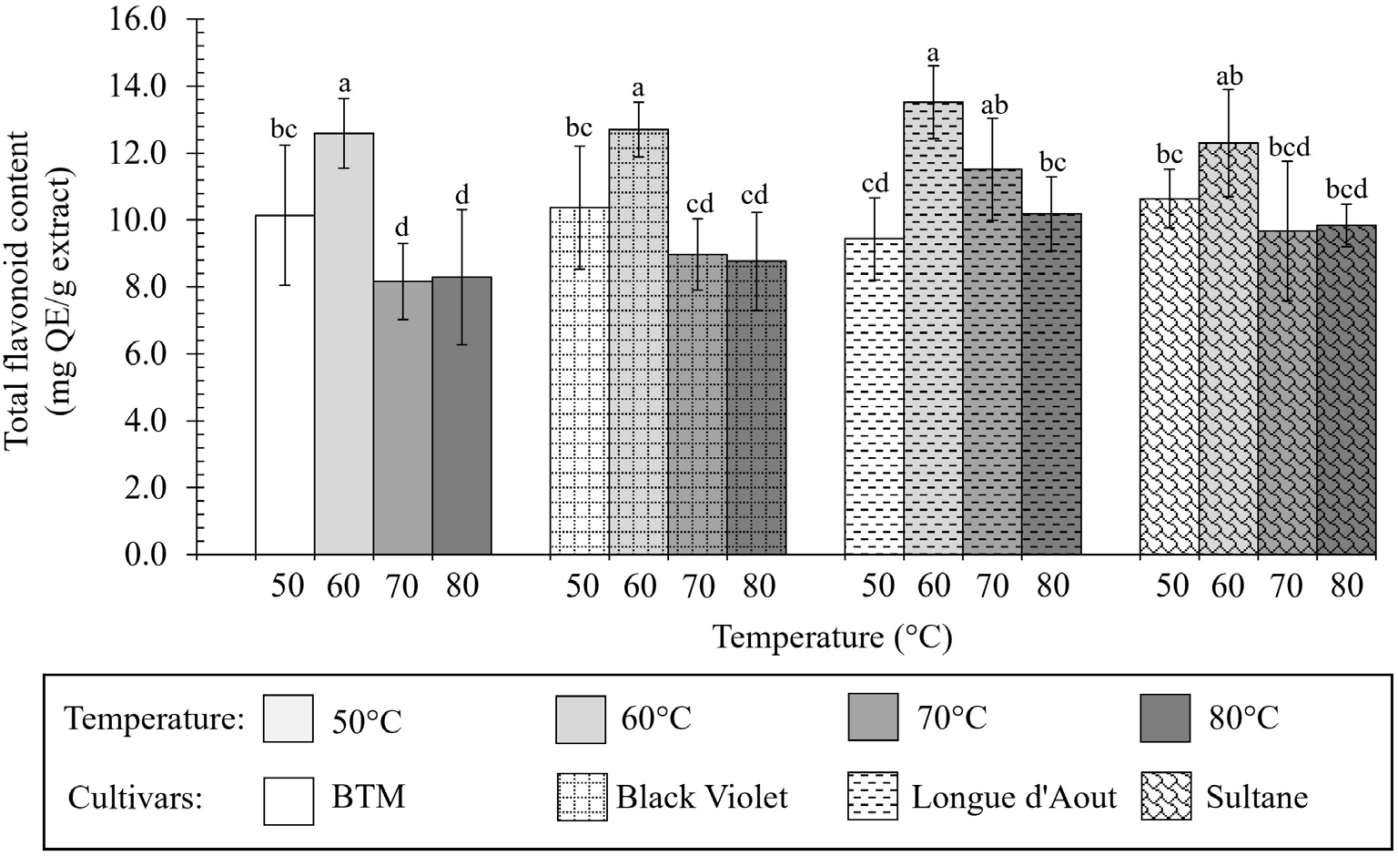
Effect of drying temperature on total flavonoid content (mg QE/g extract) of fig leaf extracts from different cultivars using water extraction. Different letters indicate significant differences (*p* < 0.05). Values are shown as mean ± SD (*n* = 3).

Total phenolic content (TPC) and total flavonoid content (TFC) of fig leaf extracts from different cultivars using water extraction as shown in Figures 3 and 4, Table 1.

**TABLE 1:** Effect of drying temperature on total phenolic content (TPC) and total flavonoid content (TFC) of fig leaf extracts.

Cultivar	Temperature (°C)	TPC (mg GAE/g)	TFC (QE/g)
BTM	50	39.61 ± 9.33^de^	10.14 ± 2.09^bc^
	60	47.61 ± 6.62^abc^	12.59 ± 1.04^a^
	70	41.79 ± 4.55^cd^	8.16 ± 1.13^d^
	80	45.22 ± 2.26^bcd^	8.29 ± 2.01^d^
BV	50	37.89 ± 10.54^de^	10.37 ± 1.83^bc^
	60	40.49 ± 3.10^de^	12.70 ± 0.82^a^
	70	32.70 ± 2.00^e^	8.97 ± 1.07^cd^
	80	35.61 ± 6.83^e^	8.77 ± 1.46^cd^
Longue d'Aout	50	37.53 ± 5.42^de^	9.44 ± 1.23^cd^
	60	53.80 ± 4.80^a^	13.51 ± 1.09^a^
	70	49.80 ± 3.76^ab^	11.51 ± 1.53^ab^
	80	47.77 ± 5.45^abc^	10.19 ± 1.11^bc^
Sultane	50	37.37 ± 3.60^de^	10.64 ± 0.87^bc^
	60	50.73 ± 7.20^ab^	12.30 ± 1.60^ab^
	70	47.87 ± 1.99^abc^	9.67 ± 2.08^bcd^
	80	47.09 ± 2.71^abc^	9.84 ± 0.63^bcd^

*Note:* Different letters within the same cultivar and assay indicate significant differences (*p* < 0.05), with letters assigned in ascending order of values. Values are shown as mean ± SD (*n* = 3).

Abbreviations: BV, Black Violet; GAE, gallic acid equivalent; QE, quercetin equivalent.

TPC varied significantly among cultivars and temperatures (*p* < 0.05), ranging from 32.70 ± 2.00 to 53.80 ± 4.80 mg GAE/g extract (Figure 3). TFC ranged from 8.16 ± 1.13 to 13.51 ± 1.09 mg QE/g extract (Figure 4, *p* < 0.01). At optimal 60°C, Longue d'Aout achieved the highest TPC (53.80 ± 4.80 mg GAE/g), comparable to Sultane (*p* > 0.05). Longue d'Aout and Sultane also showed numerically higher TFC (13.51 ± 1.09 and 12.30 ± 1.60 mg QE/g) than BTM and Black Violet (12.30 ± 0.75 and 12.70 ± 0.81 mg QE/g, TFC at 60°C). At 60°C, all cultivars achieved maximum TFC with no significant differences (*p* > 0.05). Patterns aligned between TPC and TFC, suggesting cultivar‐specific phenolic and flavonoid accumulation. The plant varieties are ranked by phenolic content from highest to lowest as follows: Longue d'Aout, Sultane, BTM, and Black Violet, respectively (Table 1).

Thermal sensitivity differed markedly between TPC and TFC compounds across cultivars, with substantial effect magnitudes (Cohen's *d* = 1.52–1.87). Elevating the drying temperature above 60°C led to phenolic degradation. Black Violet leaves demonstrated greater thermal sensitivity (Cohen's *d* = 1.62), with TPC declining from 40.49 mg at 60°C to 35.61 mg at 80°C (11.9% loss), while Longue d'Aout maintained greater thermal stability (Cohen's *d* = 0.89), with 11.3% loss to 47.77 mg GAE/g (88.7% retention). For flavonoids, Black Violet showed the steepest decline above 60°C (*p* < 0.05), decreasing 30.9% at 80°C. Longue d'Aout maintained greater stability with a 24.6% loss. BTM showed intermediate sensitivity (27.6% loss), while Sultane was most stable (23.5% loss).

Notably, TFC decline rates were lower than TPC decline rates. Flavonoids represent more thermally stable components than phenolic acids (Table 1, Petruccelli et al. 2018). Decreased phenolic content above 60°C results from thermal decomposition through dehydrogenation and oxidation reactions (Antony and Farid 2022; Oancea 2021; ElGamal et al. 2023). Phenolic compounds are characterized by hydroxyl groups attached to aromatic ring systems, which confer their characteristic antioxidant activity (Rahman et al. 2015). Hydrogen‐donating (reductive) properties from hydroxyl groups and aromatic ring systems are critical for antioxidant activity (ElGamal et al. 2023). Under elevated temperatures, functional groups undergo oxidative cleavage and condensation reactions. Aldehydes, ketones, or carboxyl groups form via reactions with atmospheric oxygen (ElGamal et al. 2023). Boukhalfa et al. (2025) reported 25%–36% greater phenolic degradation at 70°C than at lower temperatures. Anthocyanins, which accumulate in dark‐pigmented cultivars like Black Violet and contribute substantially to leaf pigmentation, are exceptionally heat sensitive. Glycosidic bonds in anthocyanins readily hydrolyze under elevated temperatures (Boukhalfa et al. 2025; Xue et al. 2024). Hydrolysis degrades anthocyanins into anthocyanidins and colorless phenolic acids (Boukhalfa et al. 2025). In contrast, hydroxycinnamic acids (e.g., caffeic acid) and quercetin demonstrate greater thermostability (ElGamal et al. 2023; Hermawati et al. 2023; Zhang et al. 2019). Quercetin and hydroxycinnamic acids naturally exist in both glycosylated and aglycone forms (Boukhalfa et al. 2025). Aglycone forms demonstrate particular resistance to thermal degradation above 75°C. This structural difference explains why these compounds maintain stability at elevated drying temperatures, where glycosylated forms degrade.

Cultivar‐specific thermal stability likely reflects genetic differences in phenolic compound composition and distribution (Table 1). Black Violet's thermal sensitivity indicates greater dependence on heat‐labile anthocyanins. Longue d'Aout's superior thermal stability suggests enrichment in thermostable phenolic compounds, particularly hydroxycinnamic acids and quercetin. Fig leaves contain diverse phenolic compounds with anthocyanins particularly abundant in dark‐pigmented cultivars (Wang et al. 2024). Petruccelli et al. (2018) reported phenolic contents ranging from 16.2 to 38.9 mg GAE/g extract in Italian fig cultivars. Notably, hydroxycinnamic acids (particularly caffeic acid derivatives) were consistently predominant phenolic class across cultivars, with significant cultivar‐specific variation (Wang et al. 2024). Cultivar selection is as critical as temperature optimization for developing functional ingredients with superior phenolic retention.

Moreover, water effectively recovers glycosylated flavonoids naturally abundant in fresh fig leaf tissues through hydrogen bonding with sugar moieties (Plaskova and Mlcek 2023). Organic solvents extract free aglycones (the non‐glycosylated forms) more effectively than glycosylated flavonoids (Li et al. 2024). These aglycone forms naturally accumulate in processed or aged fig tissues where enzymatic sugar removal has occurred. Method selection determines whether measurements reflect predominantly glycosylated or mixed flavonoid profiles. TFC across cultivars ranged from 6.78 to 13.51 mg QE/g extract, with the highest values at optimal 60°C (Table 1). Petruccelli et al. (2018) reported similar TFC values (7.30–14.80 mg QE/g) in Italian fig cultivars. Observed values align with published data, confirming consistency and broad uniformity of flavonoid content across diverse fig germplasm.

### 3.3 Antioxidant Activities

Antioxidant activities varied considerably across three assays, showing cultivar‐specific and temperature‐dependent patterns (Table 2). DPPH radical scavenging activity, expressed as IC_50_ values, ranged from 0.96 ± 0.01 to 2.93 ± 0.47 mg/mL. Lower IC_50_ values indicate higher activity. Longue d'Aout demonstrated the highest DPPH capacity at 60°C (0.96 ± 0.01 mg/mL). Black Violet showed second‐best performance at 50°C–60°C (1.03 ± 0.01 mg/mL). ABTS radical scavenging activity ranged from 8.77 ± 0.37 to 14.44 ± 0.26 mg TE/g extract. Sultane exhibited the highest ABTS activity at 80°C (14.44 ± 0.26 mg TE/g extract). Longue d'Aout and Sultane showed increased ABTS activity with elevated temperature. FRAP values ranged from 0.42 ± 0.01 to 1.28 ± 0.01 mg TE/g extract. BTM and Sultane showed maximum FRAP at 80°C, while Longue d'Aout maintained stable values across temperatures.

**TABLE 2:** Antioxidant activities of fig leaf extracts at different drying temperatures.

Cultivar	Temperature (°C)	DPPH IC_50_ (mg/mL)	ABTS (mg TE/g extract)	FRAP (mg TE/g extract)
BTM	50	1.33 ± 0.04ᵃ	8.86 ± 0.14ᵃ	0.44 ± 0.01ᵃ
	60	1.59 ± 0.07ᵇ	8.98 ± 0.70ᵃ	0.46 ± 0.01ᵃ
	70	2.88 ± 0.11ᶜ	9.38 ± 0.06ᵃ	0.55 ± 0.03ᵇ
	80	2.93 ± 0.47ᶜ	12.41 ± 0.26ᵇ	1.28 ± 0.01ᶜ
BV	50	1.03 ± 0.01ᵃ	8.77 ± 0.37ᵃ	0.42 ± 0.01ᵃ
	60	1.03 ± 0.04ᵃ	8.92 ± 0.71ᵃ	0.43 ± 0.01ᵃ
	70	1.39 ± 0.10ᵇ	9.18 ± 0.04ᵃ	0.44 ± 0.01ᵃ
	80	1.99 ± 0.12ᶜ	9.32 ± 0.38ᵃ	0.86 ± 0.02ᵇ
Longue d'Aout	50	1.01 ± 0.01ᵃ	9.59 ± 0.25ᵃ	0.54 ± 0.01ᵃ
	60	0.96 ± 0.01ᵃ	9.99 ± 0.05ᵃ	0.78 ± 0.00ᵇ
	70	1.07 ± 0.00ᵇ	12.88 ± 0.14ᵇ	1.11 ± 0.00ᶜ
	80	1.62 ± 0.02ᶜ	13.38 ± 0.11ᵇ	1.16 ± 0.03ᶜ
Sultane	50	1.17 ± 0.14ᵃ	12.20 ± 0.31ᵃ	0.85 ± 0.04ᵃ
	60	1.39 ± 0.04ᵃᵇ	12.30 ± 0.24ᵃ	1.04 ± 0.00ᵇ
	70	1.90 ± 0.09ᵇ	12.53 ± 0.06ᵃ	1.10 ± 0.01ᵇ
	80	2.39 ± 0.10ᶜ	14.44 ± 0.26ᵇ	1.26 ± 0.03ᶜ

*Note:* Different letters within the same cultivar and assay indicate significant differences (*p* < 0.05), with letters assigned in ascending order of values. Values are shown as mean ± SD (*n* = 3).

Abbreviations: BV, Black Violet; TE, Trolox equivalent.

Temperature significantly affected antioxidant activities across all cultivars, with distinct assay‐specific responses. DPPH activity showed optimal performance at 50°C–60°C across all cultivars. Elevated temperatures (70°C–80°C) decreased DPPH capacity in all cultivars, with BTM most sensitive (IC_50_ increased from 1.33 ± 0.04 to 2.93 ± 0.47 mg/mL). In contrast, ABTS and FRAP activities showed temperature‐dependent increases, particularly at 70°C–80°C. Longue d'Aout maintained superior DPPH activity at all temperatures while showing increased ABTS activity above 70°C. Sultane demonstrated maximum efficiency in ABTS and FRAP at 80°C. Black Violet showed moderate antioxidant activity with consistent DPPH performance but lower electron‐transfer capacity (ABTS, FRAP). BTM showed the highest thermal sensitivity in DPPH and FRAP assays.

The differential responses across assays reflect two distinct antioxidant mechanisms (Munteanu and Apetrei 2021). DPPH radical scavenging operates via hydrogen‐atom transfer (HAT), where antioxidant compounds donate hydrogen atoms to neutralize radicals (Kedare and Singh 2011). ABTS and FRAP assays measure single‐electron‐transfer (SET) capacity, where antioxidants donate electrons to reduce ferric or radical cations (Munteanu and Apetrei 2021). Temperature affected HAT and SET mechanisms differently. HAT capacity (DPPH) showed greater thermal sensitivity, declining significantly above 60°C. In contrast, SET capacity (ABTS, FRAP) showed temperature‐dependent increases, suggesting thermal‐induced enhancement of electron‐transfer compounds or improved extractability (Ioannou et al. 2020). The strong ABTS‐FRAP correlation (*r* = 0.90, *p* < 0.01) confirmed their shared SET mechanism (Munteanu and Apetrei 2021). DPPH's distinct pattern reflects its HAT‐dominant mechanism (Kedare and Singh 2011). These mechanistic differences explain why cultivars exhibiting superior DPPH activity (Longue d'Aout) did not necessarily show the highest ABTS or FRAP values.

Pearson correlation analysis revealed distinct patterns between phytochemical contents and antioxidant activities (Table 3). The strongest correlation was observed between the ABTS and FRAP assays (*r* = 0.90, *p* < 0.01), confirming their shared single‐electron‐transfer mechanism. TFC showed a significant negative correlation with DPPH (*r* = −0.51, *p* < 0.01), suggesting non‐flavonoid phenolics drive DPPH scavenging. TPC showed a weak correlation with DPPH (*r* = −0.05, *p* > 0.05), indicating phenolic quality rather than quantity determines DPPH activity. Consistent with this pattern, Longue d'Aout exhibited superior DPPH activity despite moderate TPC levels, suggesting phenolic composition quality rather than quantity determines antioxidant capacity. A positive correlation was observed between TPC and ABTS (*r* = 0.32, *p* < 0.05), suggesting phenolic compounds contribute to SET mechanisms.

**TABLE 3:** Pearson correlation coefficients between phytochemical contents and antioxidant activities of fig leaf extracts.

	TPC	TFC	DPPH	ABTS	FRAP
TPC	1	0.36^a^	−0.05	0.32^a^	0.20
TFC		1	−0.51^b^	0.05	0.04
DPPH			1	−0.14	−0.23
ABTS				1	0.90^b^
FRAP					1

*Note:* *N* = 48.

^a^ Correlation is significant at *p* < 0.05 level (2‐tailed).

^b^ Correlation is significant at *p* < 0.01 level (2‐tailed).

**FIGURE 5:**
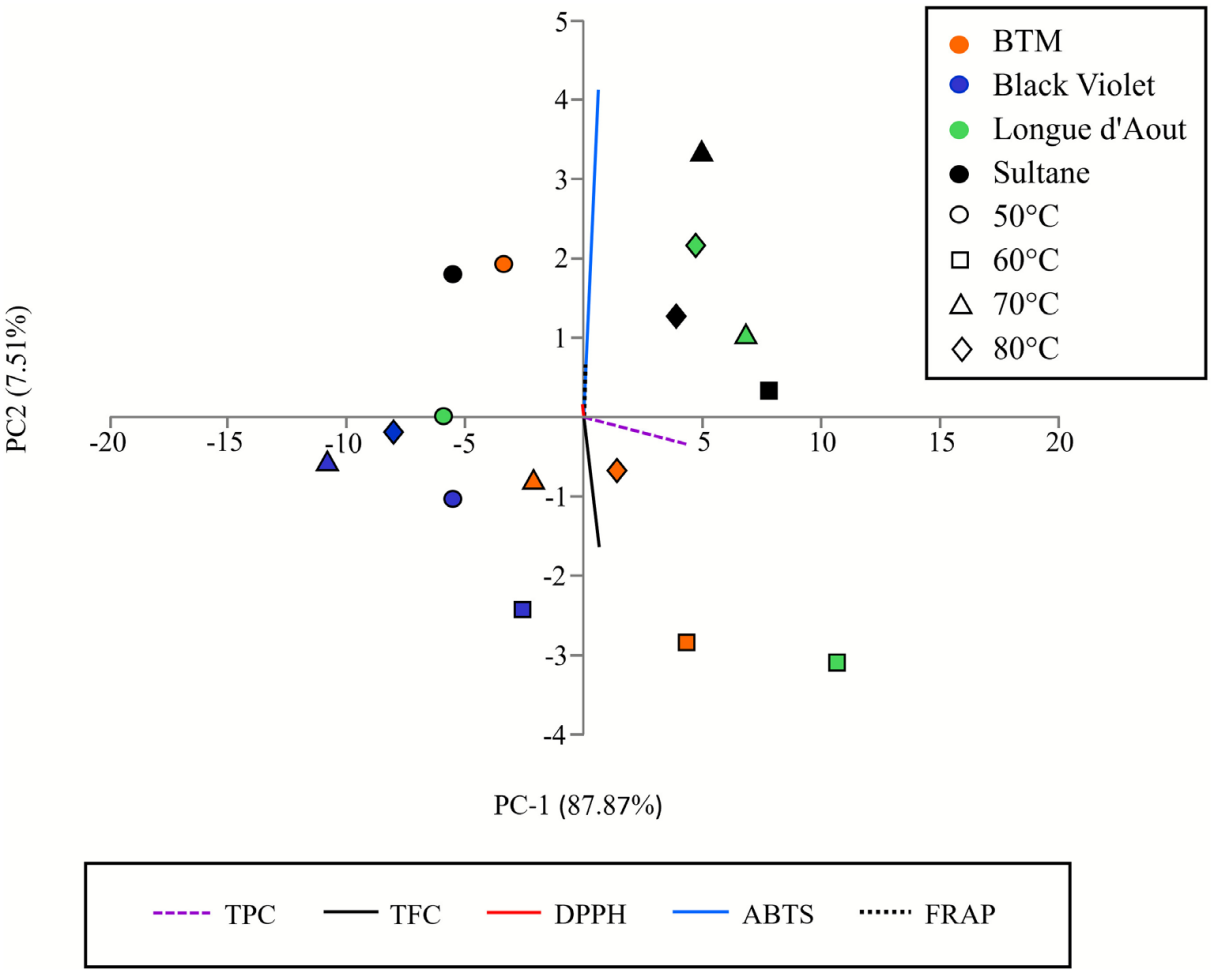
PCA biplot of biochemical parameters from fig leaf extracts. PC‐1 and PC‐2 explain 87.87% and 7.51% of variance, respectively. Points represent individual samples labeled by cultivar and drying temperature (°C). Vectors show variable loadings for TPC, TFC, DPPH (IC_50_), ABTS, and FRAP.

PCA of biochemical parameters revealed clear cultivar discrimination (Figure 5). PC‐1 and PC‐2 explained 87.87% and 7.51% of variance, respectively. Longue d'Aout extracts clustered in the positive PC‐1 quadrant, indicating superior bioactivity. Black Violet occupied the negative quadrant with lower antioxidant capacity. BTM and Sultane showed intermediate positioning. PC‐1 loadings indicated an antioxidant‐driven separation, with TPC, ABTS, and FRAP showing positive loadings. DPPH IC_50_ displayed negative loading (lower IC_50_ = higher activity). PC‐2 differentiated cultivars based on phenolic/flavonoid balance, supporting correlation patterns. PCA analysis confirmed Longue d'Aout at 60°C as the optimal processing combination.

### 3.4 FTIR Spectroscopic Analysis and Phytochemical Fingerprinting

**FIGURE 6:**
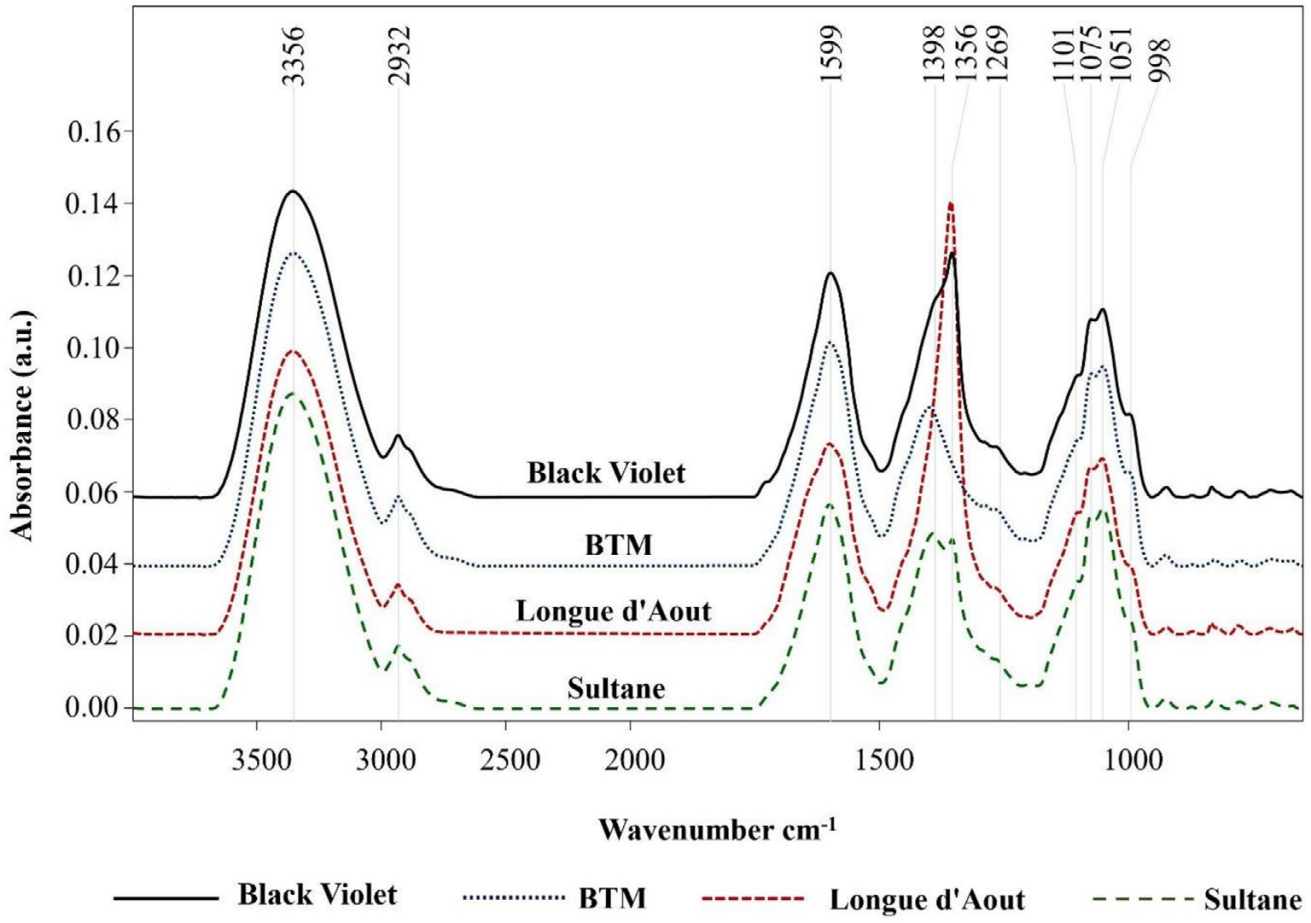
FTIR spectra of aqueous leaf extracts from four fig cultivars showing characteristic functional groups and cultivar‐specific spectral variations.

FTIR spectral analysis of aqueous extracts from four fig cultivars revealed distinct phytochemical fingerprints enabling reliable cultivar discrimination (Figure 6). All cultivars displayed common spectral features characteristic of phenolic compounds, including major peaks at ~3356 cm^−1^ (O—H stretch), ~2932 cm^−1^ (C—H stretch), ~1599 cm^−1^ (C=C aromatic), and ~1269 cm^−1^ (C—O stretch) (Wongsa et al. 2022). Moreover, the fingerprint region at 1101–998 cm^−1^ exhibited characteristic C—O stretch and skeletal vibrations of glycosidic structures, which served as distinctive biomarkers for cultivar discrimination (Kim et al. 2004).

Peak assignments were validated against literature reports of complementary analytical methods. Previous HPLC‐QTOF‐MS/MS studies identified 11 flavonoid compounds in fig leaves, including quercetin derivatives and rutin (Li et al. 2021). Similarly, literature reports of LC‐DAD/ESI‐MS^n^ analysis identified 13 distinct phenolic compounds (Shiraishi et al. 2023). In the current study, our FTIR characterization was validated against these literature compound profiles. The C=C aromatic stretch at ~1599 cm^−1^ corresponds to quercetin and other flavonoid compounds previously identified in chromatographic analyses (Li et al. 2021; Shiraishi et al. 2023). Cultivar‐specific variations included peaks at 1398 cm^−1^ (Black Violet, BTM, and Sultane) and 1356 cm^−1^ (Black Violet, Longue d'Aout, and Sultane), serving as biomarkers for cultivar discrimination (Kim et al. 2004).

Quantitative peak‐intensity analysis (Table 4) demonstrated significant cultivar differences in phenolic‐related functional groups (*p* < 0.05). Longue d'Aout exhibited the highest intensities across all phenolic‐related regions: O—H stretch (~3356 cm^−1^: 1.16 ± 0.05), C=C aromatic (~1599 cm^−1^: 0.95 ± 0.04), and C—O phenolic (~1269 cm^−1^: 0.48 ± 0.02). The hydroxyl‐to‐aliphatic ratio (I_3356_/I_2932_) exhibited cultivar‐specific patterns, with Longue d'Aout showing the highest value (4.0 ± 0.2), followed by Sultane (3.2 ± 0.1), BTM (2.4 ± 0.1), and Black Violet (2.3 ± 0.1). This ratio demonstrated a strong correlation with TPC rankings (*r* = 0.95, *p* < 0.01), confirming that FTIR spectral intensities reflect phenolic content. Similarly, the aromatic‐to‐aliphatic ratio (I_1599_/I_2932_) showed cultivar‐dependent patterns and correlated with TFC (*r* = 0.88, *p* < 0.01), validating the use of specific peak intensity ratios as quantitative descriptors.

**TABLE 4:** FTIR peak intensity ratios for cultivar discrimination.

Wavenumber (cm^−1^)	Functional group	Black Violet	BTM	Longue d'Aout	Sultane
3356	O—H stretch	0.72 ± 0.02^b^	0.85 ± 0.03^b^	1.16 ± 0.05^a^	0.98 ± 0.04^ab^
2931	C—H stretch	0.32 ± 0.01^a^	0.35 ± 0.02^a^	0.29 ± 0.01^a^	0.31 ± 0.02^a^
1598	C=C aromatic	0.64 ± 0.02^b^	0.75 ± 0.03^b^	0.95 ± 0.04^a^	0.82 ± 0.03^ab^
1398^1^	Biomarker	0.59 ± 0.02^a^	0.68 ± 0.03^a^	—	0.55 ± 0.02^a^
1356^1^	Biomarker	0.53 ± 0.02^b^	0.48 ± 0.02^b^	0.83 ± 0.03^a^	0.58 ± 0.02^b^
1268	C—O phenolic	0.28 ± 0.01^b^	0.35 ± 0.02^b^	0.48 ± 0.02^a^	0.39 ± 0.02^ab^
1100‐998	Fingerprint	0.45 ± 0.02^b^	0.52 ± 0.02^b^	0.68 ± 0.03^a^	0.55 ± 0.02^b^

*Note:* Values represent peak intensity ratios normalized to internal standard; Different superscript letters (a, b, ab) indicate significant differences among cultivars within each wavenumber (*p* < 0.05) by Duncan's multiple range test.

^1^ Cultivar‐specific biomarker peaks used for authentication; − = peak completely absent or below detection limit; Fingerprint region (1100–998 cm^−1^) values represent averaged intensities across characteristic peaks at 1100.81, 1074.87, 1050.86, and 998.32 cm^−1^, corresponding to C—O stretch and skeletal vibrations of glycosidic structures.

**FIGURE 7:**
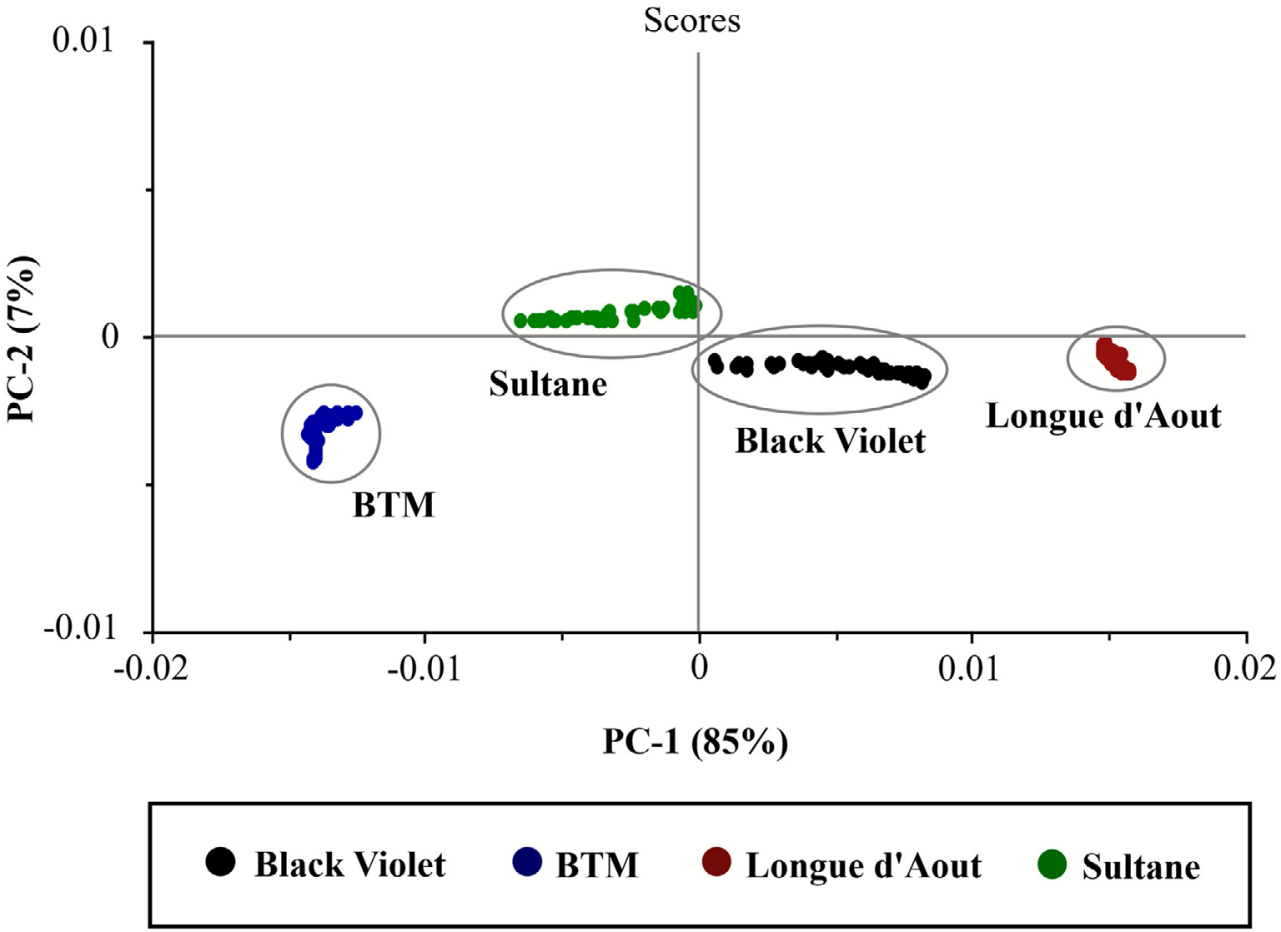
Principal component analysis (PCA) of biochemical parameters [total phenolic content (TPC), total flavonoid content (TFC), and antioxidant activities (DPPH, ABTS, FRAP)] revealed complete separation of the four fig cultivars based on their unique phytochemical profiles. PC‐1 and PC‐2 explained 92% of total variance (85% and 7%, respectively).

Principal component analysis (PCA) of biochemical parameters revealed complete separation of the four fig cultivars based on their antioxidant and phenolic profiles (Figure 7). PC‐1 and PC‐2 explained 92% of total variance (85% and 7%, respectively). The dominant PC‐1 axis was driven primarily by phenolic‐related variations (O—H and C=C aromatic stretches). Phenolic variation patterns directly reflected the cultivars' differences in total phenolic content. Clustering patterns revealed four distinct groupings correlated with phytochemical composition. Longue d'Aout clustered in the positive PC‐1 region, displaying the highest phenolic intensity values (O—H: 1.16 ± 0.05, C=C: 0.95 ± 0.04). BTM occupied the negative PC‐1 region with the lowest phenolic intensity ratios. Black Violet and Sultane exhibited intermediate positioning, reflecting their intermediate phenolic content (Table 4).

Multivariate pattern recognition captured complex biochemical differences more effectively than single‐parameter analysis. Complete cultivar separation with no cluster overlap demonstrated robust discrimination based on phytochemical fingerprints. FTIR spectroscopy combined with PCA enables rapid, non‐destructive cultivar discrimination. Such an approach offers significant advantages for cultivar authentication, quality control, and supply chain verification in fig leaf processing. LOOCV validation achieved 96.8% overall classification accuracy (48/50 correct assignments). Individual cultivar accuracy was: Black Violet 100% (50/50), Longue d'Aout 100% (50/50), BTM 96% (48/50), and Sultane 96% (48/50). Two misclassifications occurred between BTM and Sultane, likely due to overlapping phenolic profiles between these cultivars. Such high classification accuracy validates FTIR‐PCA as a robust method for rapid cultivar authentication and quality control.

### 3.5 Cell Viability and Cytotoxic Potency

**FIGURE 8:**
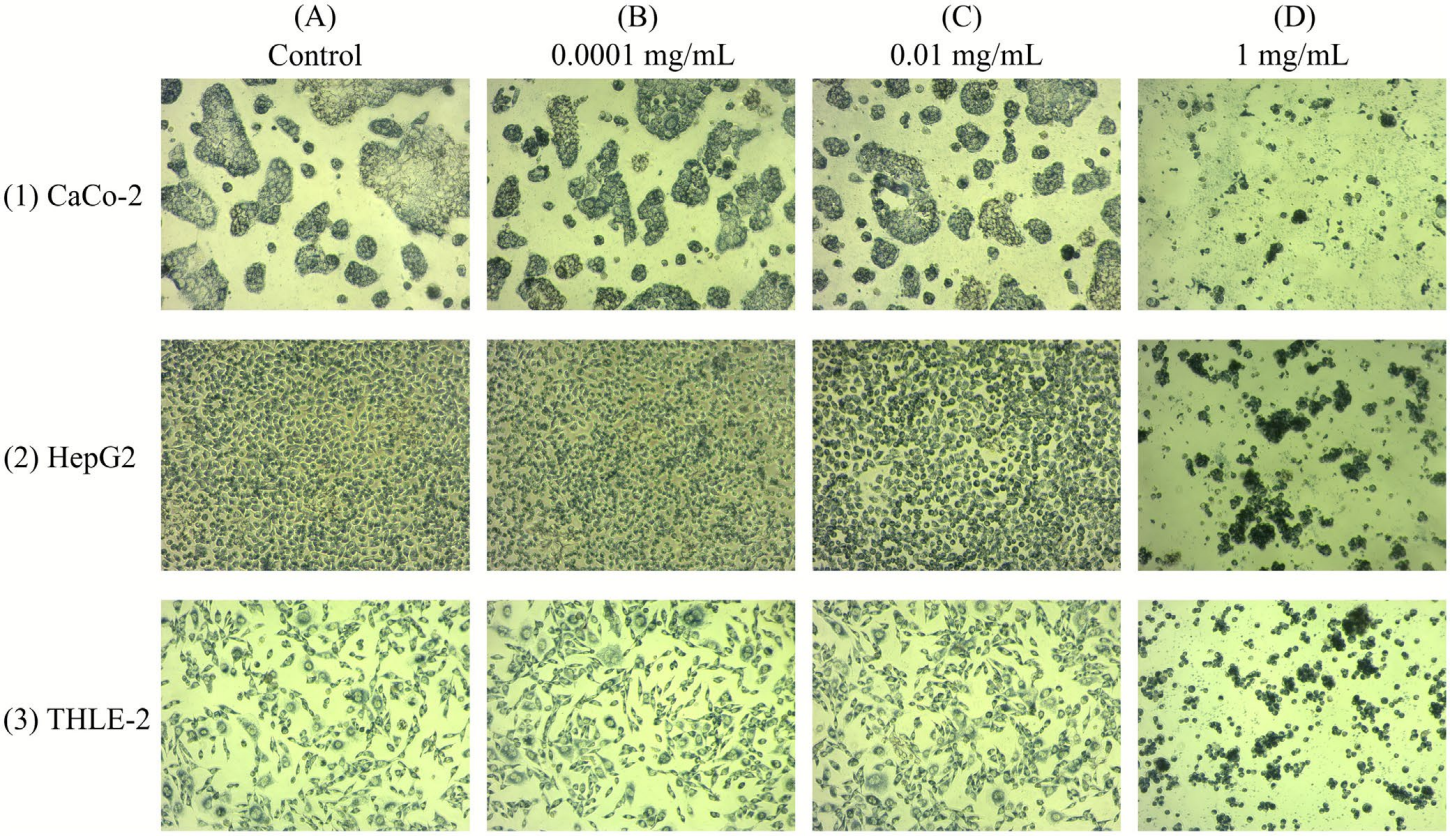
Dose‐dependent cytotoxic responses in three human cell lines exposed to Longue d'Aout fig leaf extract. Rows represent (1) Caco‐2 (intestinal epithelial cancer), (2) HepG2 (hepatic cancer), and (3) THLE‐2 (normal hepatocytes) exposed to increasing extract concentrations shown in columns: (A) Control, (B) 0.0001 mg/mL, (C) 0.01 mg/mL, and (D) 1 mg/mL. Dose‐dependent cytopathic effects were evident in phase‐contrast microscopy, characterized by progressive cell shrinkage, detachment from the substrate, and membrane blebbing at higher extract concentrations. IC_50_ values for each cell line are presented in Table 5. Values represent mean ± SD from triplicate experiments (*n* = 3).

MTT assay results revealed dose‐dependent cytotoxicity in all three cell lines (Figure 8). Longue d'Aout was evaluated based on superior antioxidant activity demonstrated in bioactive profiling.

HepG2 hepatocellular carcinoma cells exhibited the most pronounced cytopathic effects, including cell shrinkage, membrane blebbing, and extensive detachment. Caco‐2 intestinal epithelial carcinoma cells showed similar dose‐dependent changes, albeit with slightly slower progression. Normal hepatocytes (THLE‐2) remained largely intact even at maximum extract concentration, demonstrating excellent safety profile at physiologically relevant concentrations.

Quantitative IC_50_ analysis revealed cancer‐cell selective toxicity. Normal hepatocytes (THLE‐2) showed considerably lower sensitivity with IC_50_ = 15.3 ± 0.6 mg/mL, which was 2.18‐fold higher than HepG2 cells (IC_50_ = 7.0 ± 0.3 mg/mL, *p* < 0.05). Caco‐2 and HepG2 cancer cells remained viable (≥ 90% cell viability) at concentrations up to 0.01 mg/mL, while normal hepatocytes (THLE‐2) tolerated concentrations up to 0.1 mg/mL without significant reduction in viability, demonstrating a 10‐fold safety margin. These morphological and quantitative findings collectively demonstrate cancer‐specific targeting rather than generalized cellular damage. IC_50_ values and selectivity ratios for each cell line are presented in Table 5.

**TABLE 5:** Cytotoxicity profile and selectivity ratios of Longue d'Aout fig leaf extract.

Cell line	IC_50_ (mg/mL)	Nontoxic threshold (mg/mL)	Safety factor^a^	Selectivity ratio	Interpretation
Caco‐2	9.0 ± 0.5	< 0.01	900	1.70×	Some selectivity toward intestinal cancer
HepG2	7.0 ± 0.3	< 0.01	700	2.18×	Some selectivity toward hepatic cancer
THLE‐2	15.3 ± 0.6	< 0.1	1530	—	—

^a^ Safety Factor = IC_50_/practical consumption concentration (0.01 mg/mL for normal cells, estimated from functional food ingredient guidelines); Caco‐2 = Intestinal epithelial cancer cell; HepG2 = Hepatic cancer cell; THLE‐2 = Normal hepatocytes; Selectivity Ratio = THLE‐2 IC_50_/Cancer Cell IC_50_; THLE‐2 used as reference baseline; IC_50_ values are presented as mean ± standard deviation (SD) from triplicate experiments (*n* = 3).

The selective cytotoxic profile demonstrated in Table 5 and Figure 8 reveals cancer‐specific targeting of human transformed cells. HepG2 hepatocellular carcinoma cells exhibited the highest sensitivity with IC_50_ = 7.0 ± 0.3 mg/mL, followed by Caco‐2 intestinal epithelial carcinoma cells (IC_50_ = 9.0 ± 0.5 mg/mL), while normal hepatocytes (THLE‐2) showed considerably lower sensitivity (IC_50_ = 15.3 ± 0.6 mg/mL). The 2.18‐fold selectivity in IC50 values between cancer and normal cells, coupled with selective cytopathic effects in cancer cells only (Figure 8C,D), demonstrates cancer‐specific targeting rather than generalized cellular damage. Underlying cancer cell vulnerabilities account for these differential cytotoxic responses. The cancer‐specific nature of extract‐induced cell death, coupled with the extract's preferential targeting of transformed cells (Tufail et al. 2024), demonstrates the extract's potential for functional food applications. Longue d'Aout fig leaf extract thus emerges as a promising functional food ingredient with cancer‐selective potential.

The mechanism likely involves redox stress. The high phenolic content in Longue d'Aout (53.8 mg GAE/g) may cause antioxidant overload in cancer cells, which operate at higher basal ROS thresholds than normal cells (Ma et al. 2025). This exogenous antioxidant stress disrupts their precarious redox balance, whereas normal cells maintain metabolic flexibility better, allowing adaptation (Ma et al. 2025). Such redox stress activates apoptotic pathways, as phenolic compounds in fig leaves are documented inducers of intrinsic apoptosis in cancer cells (Quero et al. 2022). Mitochondrial dysfunction and caspase activation are triggered by phenolic compounds (Bakrim et al. 2022), mechanisms that preferentially affect rapidly dividing cancer cells over slowly dividing normal hepatocytes. All cancer cell lines maintained > 90% viability at ≤ 0.01 while normal hepatocytes tolerated 0.1 mg/mL (Table 5), supporting this antioxidant stress hypothesis. At lower concentrations the extract acts as a beneficial antioxidant, at higher concentrations it becomes pro‐oxidant in cancer cells.

Considering the 2.18‐fold selectivity ratio, the profile aligns with typical plant‐derived compounds (1.5–4.0 range) that are considered promising for mechanistic investigation as potential anti‐cancer agents (Calderón‐Montaño et al. 2021; Ouverney et al. 2025). Although this falls short of the > 5–10 ratios required for clinical drug development (Donnelly et al. 2025), the safety profile is substantial. Normal hepatocytes tolerate 10‐fold higher concentrations than cancer cells, substantially exceeding acceptable thresholds for safe functional food formulation. Although fig leaves have been consumed for centuries in Mediterranean regions without reported adverse effects (Badgujar et al. 2014), our findings confirm the safety profile of this plant material.

The Longue d'Aout cultivar's high phenolic content (53.8 mg GAE/g), excellent antioxidant activity, and selective cytotoxicity establish the extract as a promising functional food candidate. Natural compounds that act as beneficial antioxidants in normal physiology yet selective toxins in cancer cells represent a rarely achieved property in plant‐derived compounds. Therefore, the demonstrated dual functionality validates the extract's potential as a functional food ingredient and warrants further investigation into its bioactive mechanisms. While the findings are compelling, this study was limited by its scope. Only three biological replicates from a single geographic location (Pak Chong District) were examined, which may restrict the generalizability of results across cultivars and geographic regions. Additionally, seasonal variation in phytochemical composition was not assessed, a factor that could significantly influence cultivar‐specific bioactivity profiles.

## 4 Conclusion

Fig leaf extracts can be effectively characterized and cultivars discriminated using optimized aqueous extraction protocols (60°C drying) and FTIR fingerprinting methodology. Longue d'Aout exhibited superior bioactive content (TPC = 53.8 mg GAE/g extract, DPPH IC_50_ = 0.96 mg/mL), with bioactivity declining significantly above 60°C across all cultivars. Distinct phytochemical fingerprints of fig leaves were discriminated by FTIR‐PCA with 96.8% accuracy and complete cluster separation, validating rapid, non‐destructive fingerprinting for cultivar authentication. Cell‐based cytotoxicity assessment on three human cell lines (Caco‐2, HepG2, and THLE‐2) revealed excellent safety profiles (IC_50_ = 7–15.3 mg/mL, safety factors > 700). Notably, selective toxicity toward cancer cells was observed (2.18‐fold selectivity ratio). The FTIR fingerprinting method provides rapid, non‐destructive quality assurance and cultivar authentication, enabling scalable production of functional products. Current findings establish fig leaf extracts as promising functional food candidates. Integration of temperature‐optimized extraction, safety validation, and spectroscopic authentication establishes fig leaf extracts as functional food ingredients. However, future work should prioritize in vivo validation, quantitative mechanistic investigation of apoptotic pathways using advanced analytical instrumentation (flow cytometry, gene expression analysis, mass spectrometry), comparison of alternative extraction and drying methods, and geographic/seasonal profiling.

## Author Contributions

Conceptualization: E.V., N.T.; Data curation: S.N., K.R., W.T., N.T.; Formal analysis: S.N., W.T., O.S., N.T.; Investigation: E.V., S.N., K.R., W.T., O.S., N.T.; Methodology: E.V., S.N., K.R., N.T.; Project administration: N.T.; Resources: S.N., U.C., P.Y.; Software: W.T., O.S., N.T.; Supervision: E.V., S.N., N.T.; Validation: E.V., S.N., N.T.; Visualization: N.K., N.T., S.N.; Writing – original draft: N.T.; Writing – review and editing: E.V., S.N., W.T., N.T.

## Conflicts of Interest

The authors declare no conflicts of interest.

## Data Availability

Research data are available from the corresponding author upon request.
